# Formation of organoid-like structures in the decellularized rat testis

**DOI:** 10.22038/IJBMS.2021.58294.12948

**Published:** 2021-11

**Authors:** Mehrafarin Kiani, Mansoureh Movahedin, Iman Halvaei, Masoud Soleimani

**Affiliations:** 1 Department of Anatomical Sciences, Faculty of Medical Sciences, Tarbiat Modares University, Tehran, Iran; 2 Department of Hematology and Stem Cell Therapy, Faculty of Medical Sciences, Tarbiat Modares University, Tehran, Iran

**Keywords:** Decellularization, Extracellular matrix, Organoid, Seminiferous tubule, Testis

## Abstract

**Objective(s):**

In testis, the extracellular matrix (ECM) in addition to the supportive role for cells in the seminiferous epithelium, is also essential for the accurate functioning of these cells. Thus, using a decellularized testicular ECM (DTECM), as a scaffold for three-dimensional (3D) culture of testicular cells can mimic native ECM for studying *in vitro* spermatogenesis.

**Materials and Methods:**

The rat testis was decellularized via perfusion of 0.5% sodium dodecyl sulfate (SDS) for 48 hr, followed by 1% Triton X-100 for 6 hr, and then 1% DNase I for 1 hr. The efficiency of decellularization was evaluated by histology, immunohistochemistry (IHC), scanning electron microscopy (SEM), and MTT test. The prepared scaffolds were recellularized with testicular cells and cultured and assessed with hematoxylin-eosin (H&E) staining after two weeks.

**Results:**

Based on the H&E image, no trace of cell components could be observed in DTECM. IHC images demonstrated collagen types I and IV, laminin, and fibronectin were preserved. Masson’s trichrome and alcian blue staining revealed that collagen and glycosaminoglycans (GAGs) were retained, and the SEM image indicated that 3D testicular architecture remained after the decellularization process. Based on the results of the MTT test, DTECM was cytocompatible, and H&E images represented that DTECM supports testicular cell arrangements in seminiferous tubule-like structures (STLSs) and organoid-like structures (OLSs).

**Conclusion:**

The results showed that the applied protocol successfully decellularized the testis tissue of the rat. Therefore, these scaffolds may provide an appropriate vehicle for in vitro reconstruction of the seminiferous tubule.

## Introduction

Spermatogenesis takes place within seminiferous tubules that are supported by a basal lamina, which is a modified form of ECM that is specialized for this purpose. ECM is a rich source of proteins such as laminin, collagens, and fibronectin, also, glycosaminoglycans (GAGs) are abundant in there (1-3). ECM besides Sertoli and interstitial cells creates the main niche for germ cells (4). It has been proven that ECM of each anatomical region has a unique structure that plays a determinative role in the fate and differentiation of cells (5). Thus, the use of a native decellularized testicular ECM as a biologic scaffold is a suitable alternative for researchers to study disorders in the gametogenesis process. Biological scaffolds are produced by the decellularization of tissues. In this technique, the cells are isolated from tissues while ECM remains and is known as the scaffold (6). It has already been reported that the decellularization of the testis was successfully performed and ECM components were preserved as well. Based on previous evidence, human and mouse testicular cells survived and organoid-like structures were formed in these scaffolds but a structure similar to seminiferous tubules or testis tissue was not formed (7, 8). 

In this study, the whole of rat testis was decellularized and after characterization of DTECM, to study the behavior of special testicular tissue cells in DTECM, recellularized with rat testicular cells, cultivated for two weeks, and assessed with H&E. 

## Materials and Methods

***Animals***

The Sprague-Dawley rats were kept in a 12:12-hr light-dark cycle at 22 ± 2 °C with free access to food and water. All procedures on animals were conducted using guidelines approved by the Ethics Committee of Tarbiat Modares University (Permission No. IR.TMU.REC.1395.521). 

***Testis decellularization***

The testes were obtained from rats weighing 120–150 g in sterile conditions and transported under a laminar hood. The capsules of the testes were perforated at both ends with an insulin syringe, cannulated on one side using a surgical tube and held in place with sutures, and then decellularized under constant perfusion of the decellularization solutions at room temperature. The samples were perfused with 0.5% sodium dodecyl sulfate (SDS, Carl Roth, Germany) in deionized distilled water (DDW) for 48 hr at a rate of 0.5 cc/min using the perfusion pump, followed by 1% Triton X-100 (Sigma, Germany) in DDW for 6 hr. Next, 1% DNase I (Sigma, Germany) in phosphate buffer saline (PBS) was perfused for 1 hr in order to complete the elimination of DNA, followed by washing with 0.1% Pen Strep/PBS for 12 hr. 

***Decellularization efficiency***

Fixation of the scaffold and native tissue was performed in 10% formalin for 48 hr, followed embedding in paraffin. Sections with 5 μm thickness were stained with H&E for evaluation of decellularization process efficiency of cellular component removal. Preservation of collagens was assessed by Masson’s trichrome (Merck, Germany). Alcian blue (Merck, Germany) staining was performed for study retention of GAGs. 

***Immunohistochemistry (IHC)***

The retention of collagen types I and IV, laminin, and fibronectin in DTECM was evaluated by IHC. Sections were de-waxed and then rehydrated by alcohol gradients. For antigen retrieval, the tissue sections were heated in buffer citrate for 30 min, followed by using 10% goat serum in PBS to block the non-specific binding sites. Next, the sections were incubated with anti-fibronectin, anti-collagen IV, anti-collagen I (all from Elabscience, China), and antilaminin (Sigma, Aldrich) antibodies. Alexa Fluor 488 -conjugated secondary antibody (Elabscience, China) was applied for 60 min at 37 °C in a dark place, and images were captured with a fluorescent microscope (Olympus, type CH2). All processes were performed on intact testis as control, and finally, 0.1 µg/ml blue-fluorescent 4, 6-diamidino-2-phenylindole (DAPI, Carl Roth, Germany) was used for nuclei staining. 

***Scanning electron microscopy (SEM)***

The 3D structure of DTECM was assessed with SEM. Intact testis and DTECM were fixed in 2.5% glutaraldehyde (Merck, Germany) for 4 hr at 4 °C, dehydrated in ethanol by increasing concentration, and dried with a freeze drier. Then, the samples were covered with gold and imaged by an SEM microscope (Phenom, Netherlands). 

***DNA content analysis and DAPI staining***

To measure the amount of DNA remaining in DTECM, a QIAamp DNA Mini Kit was used according to the manufacturer’s guideline (Qiagen, Germany) for native and decellularized tissues (9). DNA concentration was measured by a NanoDrop™ 2000/c spectrophotometer (Thermo Scientific, Netherlands) at 260 nm. The section of DTECM was stained with DAPI for detection of dsDNA residuals, and the native testis was stained for control. 

***Cytotoxicity assay***

The cytotoxicity of DTECM was measured via MTT (Carl Roth, Germany) on the proliferative rate of rat testicular cells. The test was performed with the previously described protocol by Baert et al (10). 

***Isolation and culturing of neonatal rat testicular cells***

To investigate the behavior of testis-specific cells in DTECM, neonatal rat testicular cells were transplanted into decellularized scaffolds. These cells were isolated from male rat pups (1–2 days old) with three-step enzymatic digestion, 0.5 mg/ml collagenase IV, 0.5 mg/ml trypsin, and 0.5 mg/ ml hyaluronidase (all from Sigma, Germany) according to Anjamrooz protocol (11). The suspension cells were cultivated in an alpha minimum essential medium (αMEM, Bio-Ideal, Iran) supplemented with 10% FBS containing 100 U/ml penicillin and 100 μg/ml streptomycin at 34 °C in 5% CO2. 

***Preparation of support layer for tissue culture***

To this end, 1.5 g of agarose was dissolved in 100 ml of double-distilled water and sterilized to prepare agarose gel. The prepared gel was cut into pieces with 1 cm × 1 cm × 5 mm dimensions and placed in a culture plate. Gel pieces were incubated in αMEM supplemented with 10% FBS containing 100 U/ml penicillin and 100 μg/ml streptomycin at 34 °C in 5% CO2 for 24 hr. 

***Transplantation of testicular cells in DTECM***

**Figure 1:**
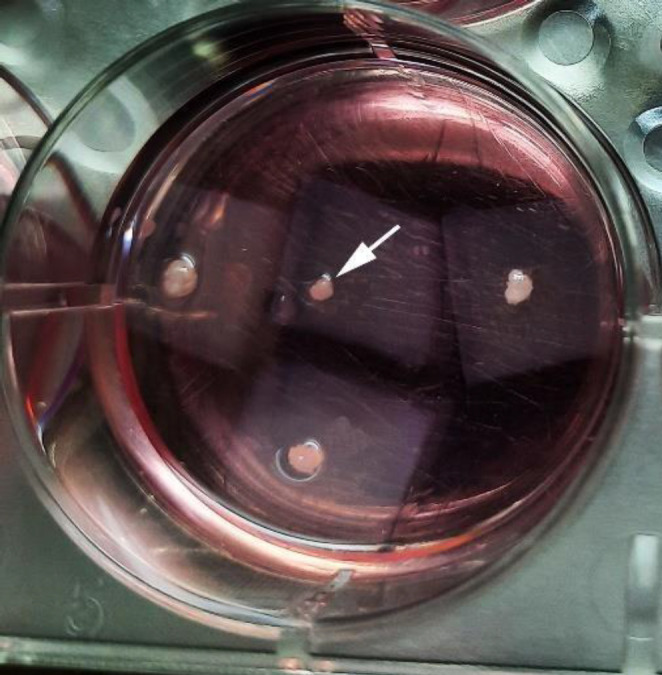
Culture pieces of Cell-loaded scaffolds on agarose gel

The efferent duct was cannulated, 100 μl cell suspension containing 1,000,000 cells was injected into seminiferous tubules. Cell-loaded scaffolds were cut into 1×1×1 mm^3^ pieces and placed on agarose gel in a 6-well plate, and then cultivated in αMEM supplemented with 10% FBS at 34 °C in 5% CO_2_ (Figure 1, arrow). The scaffolds were fixed and sectioned after two weeks, followed by performing H&E staining. 

***Efficiency evaluation of formation of STLS and OLS and comparing the diameter of these structures with that of seminiferous tubules in native tissues***

To evaluate efficiency, the percentage of the segments of DTECM with STLSs and OLSs was measured relative to all segments, and the mean diameter of these structures was compared with that of seminiferous tubules in the testes of adult and neonatal rats. 

***Statistical analysis***

Data were analyzed by one-way ANOVA tests with Tukey’s test using SPSS 15 (Chicago, USA) and presented as the mean±standard deviation, and *P*<0.05 was considered statistically significant. 

## Results

**Figure 2:**
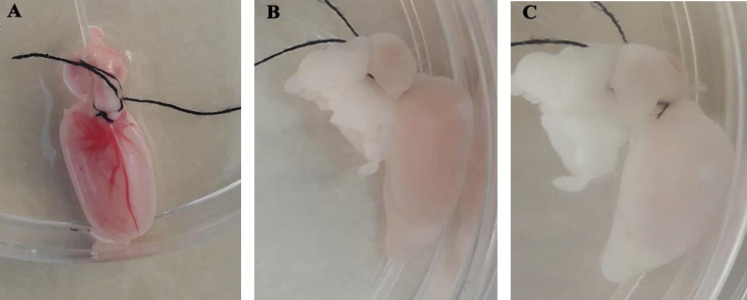
Appearance changes of rat testis during decellularization. At the beginning of the experiment, the testis was opaque and pink (A), after 24 hr it was brighter (B), and post 48 hr was completely whitish translucent (C)

**Figure 3:**
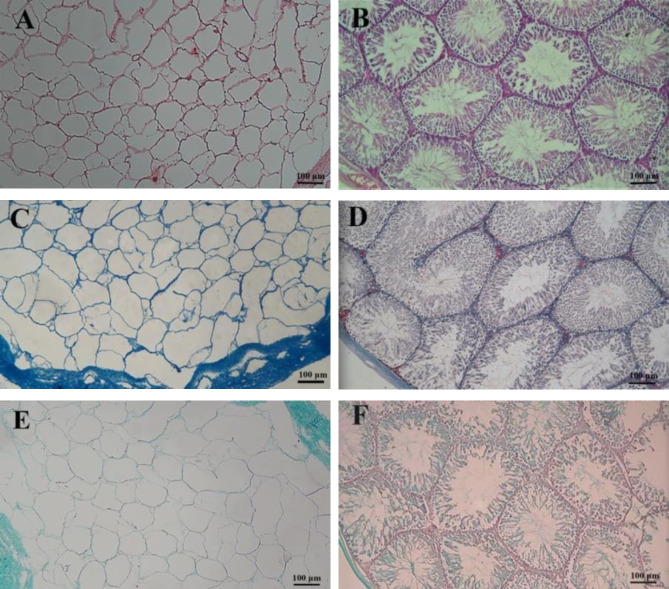
Histological evaluation of decellularized testicular ECM (DTECM). H&E staining comparison of decellularized (A) and native testes (B) exhibited the elimination of the cells and preserved extracellular matrix (ECM) architecture. Masson’s trichrome staining showed collagen fiber was preserved after the decellularization process (C) similar to the intact testis (D). Alcian blue staining demonstrated the preservation of GAGs following decellularization process (E) intact testis stained as control (F)

The external changes of the tissue during decellularization indicated that the decellularization process was successful. The intact testes were opaque and pink (Figure 2A), which were brighter and translucent after 24 hr from the beginning of the experiment (Figure 2B) and after 48 hr (Figure 2C), respectively. Histological evaluations confirmed that decellularized protocol effectively removed cellular materials while preserving ECM architecture. Based on the H&E image, the structure of the tissue was approximately preserved while there was no trace of cells and cellular components (Figure 3A). Native testis was stained as a control (Figure 3B). Trichrome staining clearly showed that the preserved collagen fibers after decellularization (Figure 3C) were similar in native testis (Figure 3D). Alcian blue staining demonstrated the preservation of GAGs following decellularization, and it was present throughout DTECM after decellularization (Figure 3E). Native testis was stained as control (Figure 3F). 

**Figure 4:**
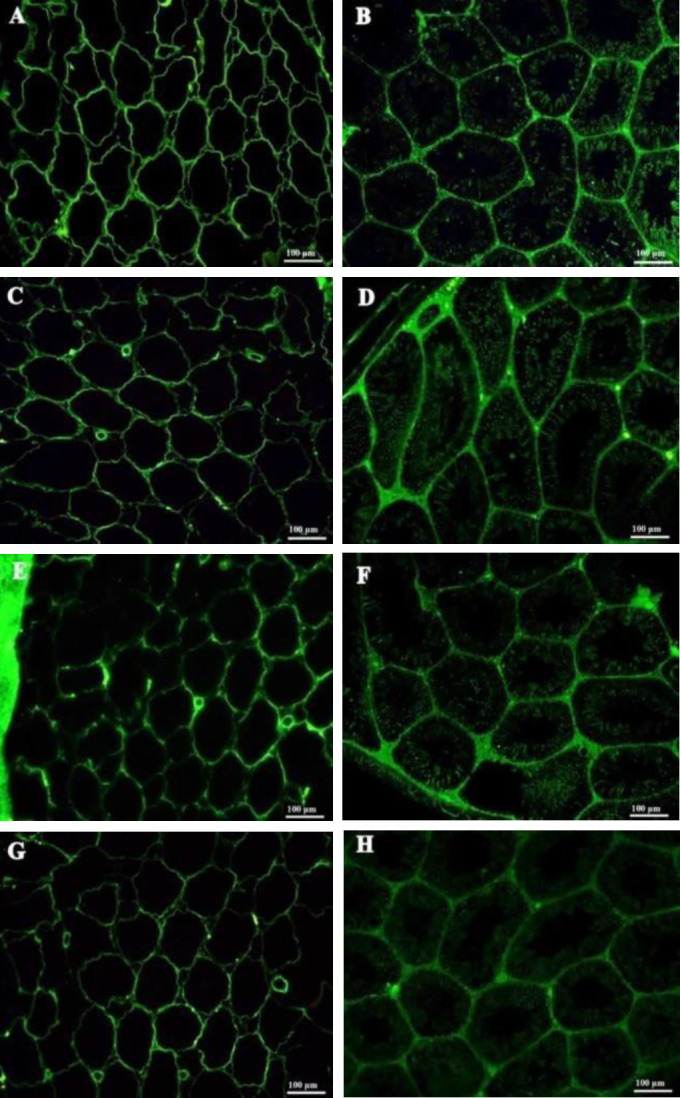
Immunohistochemistry (IHC) of extracellular matrix (ECM) proteins in decellularized testicular ECM (DTECM). Collagen type I (A and B), collagen type IV (C and D), fibronectin (E and F), and Laminin (G and H) were detected in decellularized testes (left series) with similar patterns as in native testes (right series)

Immunostaining was used for verifying the presence of four important proteins of ECM, including collagen type I and collagen type IV, laminin, and fibronectin in DTECM. Collagen types I (Figure 4A) and IV (Figure 4C) were present in DTECM with similar distributions in native testis (Figures 4B and D). The presence of collagen type IV was more pronounced in the surrounding area of seminiferous tubules and blood vessels. Additionally, cell adhesion molecules, laminin, and fibronectin were found to be present throughout the matrix of DTECM (Figures 4E and G), which was the same pattern as that of the native testis (Figures 4F and H). In general, these proteins were preserved in DTECM (left panel) with a similar pattern in the native testis (the right panel). 

**Figure 5:**
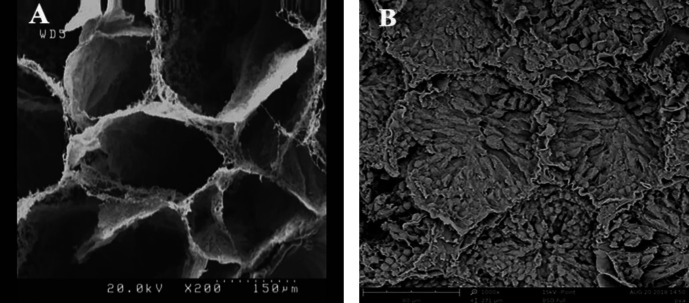
SEM image of the 3D structure of decellularized testicular ECM (DTECM). The image indicates the preservation of the 3D testicular architecture in the decellularized scaffold, seminiferous tubules are shown without any cell (A). Image of intact testis used as control (B)

The SEM image of DTECM confirmed the preservation of 3D ECM architecture and the shape of seminiferous tubules without cellular compartment (Figure 5A), and the image of the native testis was applied as control (Figure 5B). 

**Figure 6:**
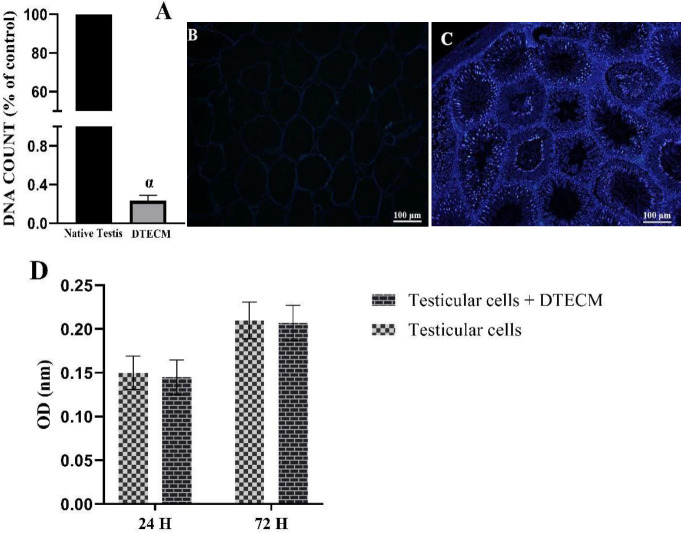
DNA content and cytocompatibility assessment of decellularized testicular ECM (DTECM). DNA quantification confirmed that the decellularization process almost removed the entire DNA content from the decellularized tissue (A). DAPI staining of the DTECM section also confirms this (B). The native tissue was stained for control (C). Optical density (OD) values, did not indicate the cytotoxic effect of decellularized scaffold on the proliferative activity of testicular cells (D). The cells kept a normal proliferation rate compared with the controls

Based on the analysis of DNA content, approximately 99% of the DNA content was removed from DTECM (Figure 6A). Furthermore, DAPI staining showed that the nucleus was not observed in the sections of DTECM, which further confirmed the efficiency of the decellularization protocol (Figure 6B), and the native testis was stained as control (Figure 6C). MTT assay represented the lack of the cytotoxic effect of DTECM on the proliferative rate of testicular cells compared with the controls after 24 and 72 hr (Figure 6D). 

**Figure 7:**
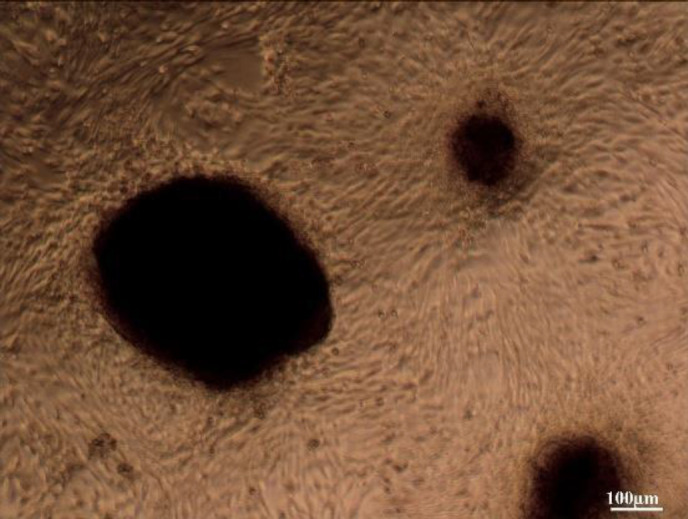
Neonatal rat testicular cells after two weeks’ culture

**Figure 8:**
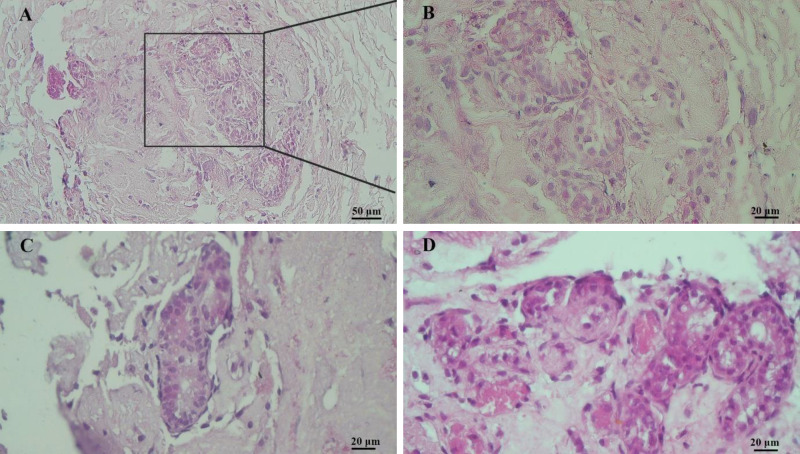
H&E staining showed testicular cells were located on the basement membrane and the seminiferous tubule-like structures (STLSs) were formed in some sections (A (B: higher magnification) and C) and the organoid-like structures (OLSs) were observed in other sections (D)

The neonatal rat testicular cells were isolated and cultured for two weeks to reach the appropriate density (Figure 7). In the next step of the experiment, the efficacy of DTECM was evaluated by transplantation of the neonatal testicular cells of the rats. The results indicated that cells were located on the basement membrane and the STLSs were formed (Figures 8A (B: higher magnification) and C) in some sections while the OLSs were observed in other sections (Figure 8D). Eventually, the scattered cells were approximately present in all sections, which confirmed the unique properties of the native testicular ECM on the management of loaded cells. 

***Efficiency evaluation of the formation of STLS and OLS and comparing the diameter of these structures with that of seminiferous tubules in native tissues***

**Figure 9:**
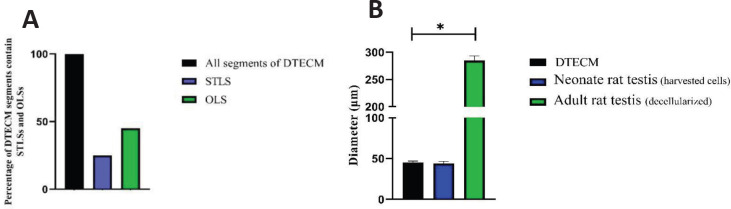
A: Data showed that 20% and 45% of the segments contained seminiferous tubule-like structure (STLS) and organoid-like structures (OLS), respectively. B: diameters of STLSs and OLSs were significantly different from that of seminiferous tubules in the adult rat testis (*P*<0.05), while not significantly differing from the diameter of seminiferous tubules in neonate rats (*P*>0.05). Data were represented by mean ± SD after three repeats

To investigate efficiency, the percentage of the segments of DTECM having STLS and OLS was measured relative to all segments, and it was shown that 20% and 45% of the segments contained STLS and OLS, respectively (Figure 9A). Which indicates the low efficiency of forming STLS. The mean diameter of these structures was compared with that of seminiferous tubules in the adult testes (decellularized) and neonatal rat testes (the harvested testicular cells). The results revealed that the diameter of STLSs and OLSs was significantly different from that of seminiferous tubules in the adult rat testis (*P*<0.05) while not significantly differing from the diameter of seminiferous tubules in neonate rats (Figure 9B). 

## Discussion

Recently, the usage of ECM scaffolds is increasing in tissue engineering for establishment of artificial organ structures in order to mimic organ functions (12), therefore, an ECM with preserved components is an ideal biomaterial for tissue engineering. This study reported a simple protocol for the preparation of a DTECM as a cytocompatible scaffold from rat testis with minimal damage to the 3D structure and tissue-specific ECM components. The most common methods for tissue decellularization including physical, chemical, and enzymatic processes (12), mainly combining multiple methods, are used to completely eliminate cells from tissues (6). In this study, the whole testis of the rat was decellularized using SDS and Triton X-100 followed by 1% D-Nase. Our results are in line with those of other studies applying SDS followed by Triton X-100 for tissue decellularization in tendon-bone, small-diameter blood vessels, and pericardium and cardiac tissues (13-16). 

The use of D-Nase in the last stage of decellularization cleans the ECM scaffold of any DNA residuals (17). More than 99% of cell DNA was removed based on DNA content analyses. The deletion of all cellular components from the decellularized scaffold causes reduced immune responses and better acceptance of host cells (18). Moreover, the maintenance of ECM structural components is essential in tissue engineering since they are necessary for communication between the cell and ECM (19). IHC images indicated important components of testicular ECM, collagens (main factor in preservation of the 3D structure), fibronectin, and laminin (important molecules for cell migration and adhesion) were retained. Other studies also confirmed that decellularization of testes via SDS and triton could retain these components (10). Thus our results indicated that the main ECM factors which are necessary for preservation of 3D structure and communication between cell and ECM were retained. 

Alcian blue staining showed that GAGs were maintained in DTECM. Moreover, GAGs affecting cell adhesion, proliferation, and differentiation can also play a crucial role in the cell outcome (20). We indicated with SEM images that the 3D structure of DTECM was preserved and other studies also reported this (21, 22). The MTT test was performed for the cytotoxicity assay of scaffolds, and the results demonstrated that DTECM was cytocompatible and had no toxic effect on cells. 

The present study examined the interaction between DTECM and specific testicular cells. The results indicated the arrangement of injected cells in STLSs and OLSs although the efficiency of STLS formation was low. Our findings are supported by the observation that reported organoid structures were formed after the transplantation of human and mouse testicular cells to decellularized testes or hydrogel scaffolds derived decellularized testes (7, 8, 23, 24), However, the observed cellular arrangement in our study was not reported in the abovementioned studies and they were unable to reconstruct structures similar to seminiferous tubules. 

Based on comparison results, there was no significant difference between the diameters of STLSs and OLSs in DTSCM and neonatal rats. Thus, the cells transplanted to DTSCM are organized in structures similar to their original position. 

The current study has some limitations. It did not examine STLSs in terms of cellular nature and cultivated in a short period, and the efficiency of the tubular-like structure was low. 

Therefore, it is recommended that other studies amend the culture system (e.g., using a dynamic culture system, growth factors, and long-term cultivation) so that to obtain a structure with architecture similar to the native tissue supporting the process of spermatogenesis. 

## Conclusion

Overall, the results of the present research showed that rat testis was successfully decellularized with a preserved 3D structure and important components of ECM. The scaffold was recellularized with testicular cells which formed STLSs and OLSs and demonstrated that the prepared scaffold supported testicular cell attachment and arrangement. 

## Authors’ Contributions

MK and MM Study design; MK Data analyzing and draft manuscript preparation; IH, MS, and MM Critical revision of the paper; MM Supervision of the research; MK, MM, MS, and IH Final approval of the version to be published.

## Funding

This study was funded by a grant from the research deputy of Tarbiat Modares University, Iran. 

## Conflicts of Interest

The authors declare no conflicts of interest. 
